# Graduate public health student learning experiences, social connectedness, and mental health during COVID-19: pedagogical implications for public health academic departments

**DOI:** 10.3389/fpubh.2024.1394034

**Published:** 2024-08-13

**Authors:** Harit Agroia, Rachel Berkowitz, Andrew Carter, Vicky Gomez, Monica Allen

**Affiliations:** Department of Public Health and Recreation, San José State University, San Jose, CA, United States

**Keywords:** learning experiences, COVID-19, public health, social connectedness, community, mental health, pedagogy, graduate education

## Abstract

**Introduction:**

The COVID-19 pandemic impacted college student learning both globally and nationally. Current literature points to decreases in social connectedness, adverse mental health outcomes, and decreased overall learning outcomes; however, there are limited findings from higher education institutions within the Bay Area, California. There are also limited studies that examine the COVID-19 impact among public health graduate students, especially to understand how the pandemic renewed their interest in the field. The purpose of this study was to investigate the effects of COVID-19 on graduate student learning experiences, social connectedness and mental health within the Master of Public Health program at San José State University in San Jose, California.

**Methods:**

We employed a convergent mixed-methods design using a survey that quantitatively assessed student learning experiences through a retrospective pre-and post-design, and a semi-structured interview guide that qualitatively assessed student learning experiences and its relationship with mental health and social connectedness using a phenomenological design. Data were collected between September 2022 and June 2023 and analysis was performed using descriptive statistics and thematic analysis.

**Results:**

A total of 22 students completed the survey and four participated in follow-up semi-structured interviews. Of the 22, 12(54%) were among the 18–29-year age group, 9(41%) identified as Asian, 21(95%) identified as female, and 9(41%) identified as a first-generation college student. When comparing survey responses, the median change in learning experiences pre and during-pandemic were statistically significant across several areas such as student ability to stay organized (*p* < 0.0001), participate actively in class (*p* < 0.001), retain course material (*p* < 0.0001), collaborate with peers (*p* < 0.0001), and maintain a sense of connection with their cohort (*p* = 0.0001) and broader campus community (*p* < 0.0001). Semi-structured interviews further revealed the following six themes: (1) Consistent faculty support; (2) Sense of community; (3) Reduced social connectedness; (4) Impact on mental health; (5) Deeper public health understanding; and (6) Facilitators and barriers.

**Conclusion:**

Educational practitioners are encouraged to offer hybrid academic programs and establish resource infrastructures that provide students with social and mental wellness support when transitioning to different learning modalities.

## Introduction

The COVID-19 pandemic resulted in significant changes globally in the personal and professional lives of many individuals, with legal requirements that prompted long standing behavioral changes such as sheltering in place, remote learning, and limited social interactions (1). In the context of higher education, evidence suggests that these widespread changes impacted learning experiences and outcomes as well as social interactions among enrolled students; however, this literature (presented herein) is largely based outside of the United States. Other existing studies focus on evaluating mental health outcomes among college students during COVID-19. To our knowledge, there are limited studies that report findings on these aspects from universities based in the Bay Area region of California and among the public health college student population specifically. At the start of the COVID-19 pandemic, the Bay Area region of California was the first in the United States to implement shelter-in-place orders. As such, these orders uniquely affected students based locally within this region; therefore, an improved understanding of the impacts of COVID-19 on public health college student experiences specifically in this region will illuminate the unique experiences among this population, especially to understand how the pandemic may have renewed continued interest in the public health field.

### Public health student response to COVID-19

In the United States, higher education schools and programs of public health provided significant contribution to the COVID-19 response (2). These entities collectively partnered with local health departments to boost surge capacity through key pandemic response functions such as contact tracing and staff training. With respect to training, students had opportunities to gain meaningful public health experience through practical, service-learning and field experiences (2). A major study examining the impact of this important partnership found that 67% of public health schools that were part of the national organization, Association of Schools and Programs of Public Health and Council on Education in Public Health, partnered with health departments for surge capacity, 63% supported contact tracing, and 37% participated in training opportunities (2). While this provides evidence related to the student contribution to the COVID-19 response, there is a lack of understanding on COVID-19’s impact on public health college students’ continued interest in the field.

### Learning experiences and social connectedness during COVID-19

Evidence also suggests that COVID-19 generally impacted student learning experiences and outcomes both positively and negatively. Among the positive outcomes reported from the pandemic, noteworthy outcomes included having more time for other professional development activities and the remote option offering flexibility to learn from any location (3–5). Given that pursuing graduate studies is a personal choice for many, students in the Philippines had higher expectations of their learning environment, including from faculty and their teaching methods, as well as feeling an overall sense of belonging (6). In China, the learning material was found to be a significant factor for engagement, even in an online platform, as the content itself facilitated interest and learning among students (7). When these expectations are met, there is a positive correlation with overall satisfaction with an academic program (6, 8, 9). This highlights the role of faculty in ensuring a successful transition to online learning during a phenomenon like COVID-19 where evidence shows that faculty who receive training on how best to deliver educational content in an online learning environment can improve student engagement (10).

Suboptimal impacts on learning experiences were exemplified in Arantes do Amaral et al.’s study (4) among Brazilian graduate students to understand how the pandemic affected learning outcomes. They found that being sheltered in place produced adverse learning outcomes among students since many students reported experiencing digital fatigue and difficulty grasping material through online conferencing platforms. In Brazil, China and Europe, students also reported issues with using new learning technologies (4, 8, 11). These negative experiences are also observed when understanding student social connectedness during the pandemic. Al-Mawee et al. (3) employed a case study to understand student experiences with distance learning during the COVID-19 pandemic using an online survey. Their findings showed that students had overall less social interaction with their peers. Other researchers also found that this sense of social connection was connected to student perceptions and feelings of openness in being able to connect with their peers in a way that helped to develop a sense of community (9, 12, 13). Student levels of social connection may also be connected with their overall level of human interaction either with other students or with faculty, such that decreased human interaction means less engagement with course learning material (14). Understanding learning experiences, outcomes, and social connectedness among students in public health specifically may provide deeper insight into how the COVID-19 pandemic renewed student interest and engagement in the field of public health.

### Mental health impacts from COVID-19

Current mental health research related to the impact of the COVID-19 pandemic on college students’ mental wellbeing used existing assessment scales, such as the PHQ-2 and GAD-2 (15) to determine how mental health outcomes shifted among students before and after the transition to online learning. A handful of studies also incorporated questions to assess similar mental health outcomes in surveys or semi-structured interview guides. Son et al. (16) surveyed 195 graduate students to understand the effects of COVID-19 on mental health and found that 71% indicated heightened stress and anxiety during the outbreak, 89% reported difficulty concentrating, and 86% reported decreased social interactions and academic performance. Students from universities based in New York and New Jersey participated in a survey (*n* = 4,714) to understand academic, financial, and COVID-19-related stressors that impacted their learning (17). Results showed that the pandemic adversely affected their mental health overall, with most stating feeling down, depressed, hopeless and socially isolated during this time. Chrikov et al. (15) also conducted a similar study to understand mental health outcomes among more than 30,000 undergraduate and 15,000 graduate students from different universities participating in UC Berkeley and University of Minnesota’s Student Experience in the Research University (SERU) project. They found that adverse mental health outcomes were higher among students overall compared to pre-pandemic, and that those who struggled with adapting to remote instruction technologies were more likely to experience worse outcomes compared to their peers. It was also noted that students from different minoritized groups, such as low-income students and students of color, had poorer mental health outcomes (15). These combined results are consistent with findings from other studies that examined similar mental health outcomes resulting from the transition to online learning during the COVID-19 pandemic (13, 18, 19).

Specifically among students enrolled in health-related degree programs, there is limited research on mental health that focuses on the public health student population. There is, however, research from the field of nursing such as Rosenthal et al. (20) study that focused on understanding how mental health impacted nursing graduate students during the pandemic where similar health outcomes were observed. This is one of a limited number of studies that discusses mental health among graduate students from health-related academic programs; most current studies only focus on nursing programs. While Rosenthal et al. (20) study found similar mental health outcomes as those that were aforementioned, it is important to note that each academic training program is structured differently and has a variety of requirements. In many public health degree programs, graduate students do not have requirements to complete clinical hours but they may be required to complete a fieldwork experience that may introduce varying levels of complexity and stress to the student’s workload depending on their fieldwork placement.

### Significance of research

The purpose of this study was to understand the extent to which public health graduate student perceptions of learning experiences, social connectedness and mental health were impacted by the COVID-19 pandemic. We explored the perspectives of graduate students enrolled in the San José State University Master of Public Health (MPH) program, campus-format, located in San José, California. We sought to understand these factors to identify actionable pedagogical implications that directly inform future academic curriculum, instruction, and fieldwork experiences in this program.

## Materials and methods

### Setting

This study was conducted within the San José State University MPH program, housed within the Department of Public Health and Recreation. The MPH program has been accredited by the Council on Education for Public Health since 1974 and offers an MPH degree with a specialization in Community Health Education. Students are able to apply for one of two degree format options: (1) campus and (2) online. Students in the campus program have opportunities to participate in a range of extracurricular activities, such as joining student clubs, visiting the student wellness center, and participating in intramural sports. In February 2020, the campus program was shifted to online to comply with local shelter in place orders, resulting in 100% of campus-based classes shifting to online format using Zoom technology. Students resumed in-person classes in a phased approach starting in Fall 2022 (August 2022) and through the Spring 2023 (January 2023) semesters.

### Study design

This was a convergent mixed-methods study to understand the combined effects of COVID-19 on graduate student learning experiences, social connectedness, and mental health. The quantitative aspect of this study implemented a retrospective pre-and-post research design using a survey instrument. The survey was followed by the qualitative aspect which employed semi-structured interviews using a phenomenological approach. Phenomenological research is a type of qualitative design that aims to obtain a rich understanding of participant life experiences as they relate to a particular phenomenon (21). In our study, this phenomenon was defined as the COVID-19 pandemic, where we recognized that students who experienced this phenomenon were required to transition to online learning and then transition back to in-person learning once government safety laws were rescinded. Given this, students had a unique experience with COVID-19, which we sought to explore further through a phenomenological approach.

Through our design, we investigated the following research question: How did the transition to online learning impact campus student learning experiences, social connectedness and mental health? This included the following sub questions:

1. How did campus student learning experiences differ between pre- and post-online learning transitions?
2. How do public health graduate students describe their learning experiences, levels of social connectedness and mental health during the transition to the online learning environment?
3. How did these experiences affect their continued interest in the public health field?

This study was approved by the San José State University Institutional Review Board.

### Procedures

We used a stratified sampling strategy to identify and email students enrolled in the campus-format MPH Program during the 2017–2020 academic years who first transitioned to online learning and then back to campus learning during COVID-19. Eligible participants must have been attending classes on campus before the pandemic started. We also posted recruitment flyers on our social media platforms that contained a link to a survey. This survey contained screening questions to determine eligibility for study participation.

Interested students completed the screening questions and, if eligible, reviewed a consent notice and completed the quantitative survey. The survey took approximately 15–20 min to complete and assessed student demographics and learning experiences before and during pandemic. At the end of the survey, participants were asked if they were interested in being contacted by a member of our research team to participate in a follow-up qualitative interview. For participants that opted to participate in the interview, they reviewed a separate consent form and were then scheduled an interview with either the third or fifth author. The interviews took approximately 45–60 min. Individuals who completed the survey received a $20 gift card incentive. For those participating in the semi-structured interview, they received an additional $30 gift card.

### Data collection instruments

#### Quantitative data collection—survey

The quantitative survey was administered between September and December 2022. The survey contained four screening questions to assess eligibility, 11 demographic questions and 10 questions with sub questions to assess student learning experiences, social connectedness, mental health, and overall benefits and barriers to transitioning to the online learning environment. These 10 questions were grouped into pre and during-pandemic learning experiences, with the first five focusing on pre-pandemic learning and the last five focusing on during pandemic learning in the online environment. Example questions included “BEFORE the COVID-19 pandemic, what was your experience taking any online courses as part of an academic program?” and “BEFORE the COVID-19 pandemic, what was your comfort level with taking any online courses as part of an academic program?” We also included 5-point Likert scale sub-questions where students indicated their level of agreement with a set of phrases such as, “BEFORE the COVID-19 pandemic…I actively participated in class.” or “BEFORE the COVID-19 pandemic…I felt connected to the SJSU MPH community.”

#### Qualitative data collection—semi-structured interview guide

The qualitative interviews followed the quantitative survey shortly thereafter from January–June 2023. The semi-structured interview guide was developed in alignment with the stages of transcendental phenomenology (21) and contained a total of 14 questions to understand student learning experiences and their relationship with mental health and social connectedness (Table 1). We also included an emphasis on barriers to understand what students reported as barriers to achieving an optimal learning experience. Example questions included “Please describe your learning experiences generally (while enrolled in the SJSU MPH program) prior to the pandemic (prior to March 2020)?” and “Have you noticed a difference in your levels of social connection pre vs. during the pandemic?” A copy of the full interview guide can be found in Appendix 1.

**Table 1:** Example interview questions aligned with the stages of transcendental phenomenology.

Transcendental phenomenology stages	Understanding learning experiences	Understanding social connectedness	Understanding mental health
Transcendental (bracketing)	Please describe your learning experiences generally prior to the pandemic.	Have you noticed a difference in your levels of social connection pre vs. during the pandemic?	From your perspective, has the COVID-19 pandemic affected your mental health?
Phenomenological	Did the pandemic bring new insights to how you think about public health issues or how these issues can or should be dealt with?	How would you describe your current state of maintaining social connection?	How has the transition to online learning affected your mental health?
Imaginative variation (free variation)	To what extent did going into the pandemic renew or broaden their interest in public health?	What strategies can you use to prevent feelings of isolation and exclusion?	What resources have you found to be most beneficial during the pandemic?

### Data analysis

**Figure 1:**
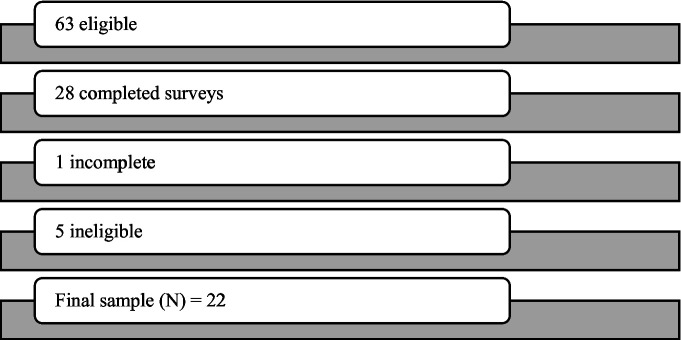
Cohort diagram.

To analyze the survey responses, raw data were first exported from Qualtrics into Microsoft Excel and reviewed for completeness and eligibility. All incomplete and ineligible response data was removed from the final dataset (Figure 1). The final dataset was then analyzed using descriptive statistics in SAS that displayed overall participant demographics (Table 2) and a comparison of overall pre and during COVID responses (Table 3). The comparison data were analyzed using the Wilcoxon signed rank test.

**Table 2:** Self-reported participant demographics.

Variable	Count (*n* = 22)	Percent of total
Age		
18–29	12	54%
30–39	7	32%
40–49	1	5%
50–64	2	9%
Race*		
Asian	9	41%
Black, African American, or African Ancestry	1	5%
Native Hawaiian or Pacific Islander	1	5%
Some other race	1	5%
White	5	23%
Ethnicity*		
Hispanic or Latino/a/e/x	10	45%
Gender identity		
Man/Male	1	5%
Woman/Female	21	95%
International student		
No	20	91%
Yes	2	9%
First generation student		
No	13	59%
Yes	9	41%
Average MPH course load		
10 or more units per semester	9	41%
4–6 units per semester	7	32%
7–9 units per semester	6	27%

Total does not equal 100% because some students specified either one race or ethnicity category but not both.

**Table 3:** Comparison of learning experiences before and during COVID-19.

	Before COVID-19 (Median, IQR)	During COVID-19 (Median, IQR)	Wilcoxon signed ranks test *p*-value
A…it was easy to stay organized and on top of my schoolwork.	4 (4,5)	4 (3,4)	<0.0001
B…I actively participated in class.	4 (4,5)	3 (3,4)	0.0012
C…I enjoyed attending class *in-person/online*	4.5 (4,5)	3 (2.5, 4)	0.0015
D…I felt that I retained course material well during my *in-person/online* classes.	4 (4,5)	3 (2,4)	<0.0001
E…I was motivated to do well in school.	5 (4.25, 5)	4 (3,4)	<0.0001
F…I was able to collaborate effectively with my peers for coursework.	5 (4.25, 5)	3 (2.25, 4)	<0.0001
G…I felt connected to the SJSU MPH community.	4.5 (4,5)	3 (2, 3.75)	0.0001
H…I felt connected to the broader SJSU community.	3.5 (3,4.75)	2 (1,2)	<0.0001
I…I enjoyed participating in extracurricular activities at SJSU (*N* = 18)	3.5 (3,4)	2 (1.25,3)	0.0098
J…I was doing well academically (e.g., my grades, how much I retained from each class)	5 (4,5)	4 (4,5)	0.0313

Participant interview data were analyzed using codebook thematic analysis (22). A codebook was created to establish an initial theme of codes based on the semi-structured interview guide. All recorded interviews were then transcribed verbatim and coded by two members of the research team. The data were then grouped by code and analyzed to identify themes. The first and third authors reviewed and finalized these themes for inclusion in our results.

## Results

A total of 22 students completed the survey (response rate = 35%) and four participated in follow-up semi-structured interviews. Of the 22, 12(54%) were among the 18–29 year age group, 14(64%) identified as Asian or White, 21(95%) identified as female, and 9(41%) identified as a first-generation college student (Table 2). These demographics are similar to those reported within the broader MPH program where a majority of enrolled students are among the 18–29 year age group (63%), identify as Asian or White (71%) race, and as female gender identity (80%).

When comparing median survey responses, there was an overall decrease in learning experiences when compared pre and during-pandemic (Table 3). Specifically, students reported a greater decrease in being able to stay organized and on top of their schoolwork (*p* < 0.0001), actively participate in class (*p* < 0.001), and retain course material (*p* < 0.0001) during the transition to online learning. Students also reported decreases in their overall motivation to do well in school (*p* < 0.0001) and academically (*p* < 0.031). With respect to their level of social connectedness and sense of community, students indicated having less opportunity to collaborate effectively with their peers (*p* < 0.0001) to complete their schoolwork, and generally felt less connected to both the SJSU MPH (*p* = 0.0001) and broader SJSU (*p* < 0.0001) community during the transition to online learning. Students also reported being less involved in extracurricular activities during this time (*p* < 0.009) (Table 3).

Semi-structured interviews offered greater context to these initial survey results, where thematic analysis resulted in the creation of the following six themes: (1) Consistent faculty support; (2) Sense of community; (3) Reduced social connectedness; (4) Impact on mental health; (5) Deeper public health understanding; and (6) Facilitators and barriers. These themes are described and discussed below.

### Consistent faculty support

All participants praised faculty for their support before and during the pandemic, especially while adjusting to a new “normal” learning environment. Participants were further appreciative of the number of faculty from diverse sociocultural backgrounds and experiences, who fostered a relatable and inclusive learning environment. Some participants indicated that faculty support never wavered throughout the pandemic; rather, there were notable shifts in the way this support was provided during the pandemic. For example, faculty continued to offer office hours, but switched modalities to remote platforms during the transition to online learning.

Participants shared:

So going into it, I knew that there was the potential of being taught by diverse and really well-rounded individuals, professors, who had a vastly different lived experience than I did and would be able to apply that into their teaching style.

And while, yes, she focused on the academic part because that’s what we were meeting for, she also took the time to kind of check in with me as a person and see how I was dealing with everything. And she even asked me, I remember, about, “What was your study environment looking like?”

I did not feel like because of what happened, all of a sudden, my faculty did not care or did not support me in the same way or the teaching went downhill or anything like that.

These reflections highlight the overall positive faculty support that the students received during the COVID-19 pandemic, where faculty were thoughtful in sharing their lived experiences during their courses and proactively worked with students to adjust their study environment for improved productivity.

### Sense of community

Most participants stated that their sense of community within the MPH program shifted before and during the pandemic. Some indicated that in-person classes simply made it easier to collaborate while online learning felt more isolating. Students could simply turn to their peer’s desks while in the same classroom together, whereas that was impossible to do in a virtual classroom. As a result, participants stated having to be more intentional and creative to find ways to work together with their peers in an online environment overall.

When reflecting on pre-pandemic experiences, one participant stated:

Having that safety net of folks around me was so helpful. Even if I was stuck in class and I thought I would figure it out and then afterwards I needed to reach out for help, I could ask my classmates because I kind of already trusted them.

When reflecting on how these experiences changed during the pandemic, one participant stated:

A lot of Zoom, a lot of outside gatherings six feet apart lawn chairs style, phone calls. I would make frozen cookies and drop off frozen cookie dough to people’s houses. And so I think that we just found ways. Those are the specifics of things that we would do. But yeah, just dropping little things off to each other.

These excerpts illuminate the value that students place in being able to work closely with their peers to do well in their academic program. There is importance in establishing an initial in-person connection with classmates in order for students to feel comfortable contacting them after class if they need more help.

### Reduced social connectedness

For students, knowing their cohort members before the transition helped sustain relationships and maintain a sense of social connectedness. However, students that were in classes with members from a newer cohort found it hard to connect with them due to cameras not being on and varying participation levels. Participants overall found it harder to brainstorm with their peers on the online platform.

Participants stated:

I did not talk to anybody outside of the group that I was already in. And I think I felt disconnected in that sense. And I felt that I could not really visit anybody’s office hours either. Even as a TA, I would visit all of the professors. I would visit the students, or the students would visit me into my office hours too. So I felt very disconnected in that sense.

I would say a little bit on the downside, especially not being able to see my classmates, not being able to interact with them. But I knew there were some courses where during the first 5 min of the class time, we were given breakout room time so that we can talk to each other about one simple question.

It was lonely and it was a lot harder.

Overall, students felt much more disconnected from their peers in the online learning environment since there were fewer opportunities to have in-person, human interactions with others.

### Impact on mental health

This section saw mixed responses from participants. A couple of participants stated experiencing positive mental health outcomes after the online transition which was attributed to the opportunity to have more time at home and with their family members. However, others reported feeling more isolated and had reduced motivation to do well in their classes.

Participants stated:

I kind of lost a lot of motivation to be a star student that I knew I could be because everything was just so weird and off and disconnected and there were bigger things in life to worry about than getting all A’s.

I do not know if it necessarily-- it made an impact, but I do not think it made a negative impact either. And I only say that because I think pre-pandemic, I was on the borderline of being burnt out too. And because COVID happened, it kind of gave me a breather room too. It was okay for me to stay at home and do nothing sometimes, or it was okay for me to not go out and to not socialize, too. And I think it did not affect me in a negative way where most people did.

### Deeper public health understanding

All participants agreed that COVID-19 brought greater awareness of the impact and complexities of working in public health.

Participants stated:

This was like a world crisis that we are kind of in right now. But it really shifted my mindset onto something that was all in textbook. It was all theoretical. And then going through a lived experience and seeing it firsthand how it affected everyone really shifted my mindset.

And so he switched gears to really use COVID as a real-life example in our work. And a lot of the professors did that. They used COVID as an example because up until now-- or up until a few years ago, we–me, had not lived through such a public health crisis. And thankfully, it brought a lot of light to the value that public health brings for the general population. And seeing it, unfortunately, in a crisis allowed me to get a clearer picture of what public health looks like in life, in society.

This highlights one positive aspect of the online learning transition in that public health students obtained a richer sense of public health practice and how academic concepts are implemented in a real-world setting. The pandemic also provided students with a sense of how much impact public health practitioners can have in the community.

### Facilitators and barriers

Most participants stated that having more time at home, less commute time, increased time with family, and linkage to campus resources all facilitated better learning experiences during the pandemic. A major barrier was reported as less opportunities for networking or relationship building with others on campus.

Participants stated:

That is something the department could have done, having some coffee things or coffee social or something just to check in with each other kind of thing. I know there were town halls where we were specifically talking about the feedback for the program. But just like meeting as a community together might have been something that I would have cherished, I guess.

I think that really sharing all of San José State’s resources with folks as they come back online. Because I’m thinking childcare and basically anything and everything that can help folks that would be doing these things at home now need to be coming in person.

## Discussion

Our results showed that graduate public health students experienced negative and significant impacts to their student learning experiences, sense of social connectedness, and mental health during the pandemic when compared to pre-pandemic. Qualitative results further revealed reasons for these significant differences and allowed the researchers to understand barriers and facilitators to improve upon these outcomes to inform future learning platform transitions. These qualitative results can be explained by Albert Bandura’s Social Learning Theory which explores how individual cognitions are informed by social observations and how these cognitions ultimately influence learning experiences (23). A main concept of the theory is observational learning; this concept argues that individuals learn and build their own skillsets by observing others (23). Those that are observed in the process of learning are seen as positive role models that promote optimal behaviors in public health. In our study, student expressed a reduced sense of social connectedness during the pandemic compared to pre- pandemic given less human interaction with their peers and other faculty, and less opportunities to connect with their cohort during campus activities. Students also shared during pandemic learning experiences declining where they were less motivated to do well in school and did not perform as well academically compared to pre-pandemic. In the context of the Social Learning Theory, these pandemic-driven changes may demonstrate how less opportunities to observe others through social interaction negatively affected learning outcomes during the COVID-19 pandemic. Bandura also highlights how positive role models in an educational space can specifically enhance an individual’s sense of belonging (23); in our study, students expressed a reduced sense of community in the MPH program under the COVID-19 shelter-in-place orders. This may further affect their perceived similarity (23) with their cohort mates as this shared group membership facilities a sense of relatability with their peers, promotes continued interest in the public health graduate learning experience, and can improve motivation to perform well academically. When students feel connected to their peers and feel a sense of community, they may also have an increased likelihood of achieving success in their academics and feel that success is more attainable than not (23).

While our quantitative results were overall aligned with existing literature, qualitative results provided unique insights into areas that were not previously investigated. First, we gained valuable insight from students regarding the extent to which the pandemic renewed their interest in public health. Students stated better understanding the complexities of the public health domain, and forming a greater appreciation for the work that public health professionals conduct during an emergency response. This may further enhance learning outcomes among students who can firsthand witness public health work in conjunction with the academic course content they were learning about in their virtual classes. Second, our participants shared valuable recommendations on what public health academic departments could learn about the pandemic, and what resources would be most beneficial to improve upon issues related to student mental health to support the transition back to in-person classes. These resources, such as childcare programs for students who did not otherwise have to seek out this program while studying from home (24), could alleviate stress and anxiety that comes with another change to their learning environment, prompted by the COVID-19 pandemic. Finally, we observed an overall decrease in learning experiences during the COVID-19 pandemic within all course load groups and especially among students that identified as 18–29 years of age given that this age group was predominantly represented in this study. Students also qualitatively affirmed how these learning experienced were impacted, much of which was attributed to the decreased sense of community and social connectedness, as well as from a mental health perspective.

### Pedagogical implications

Higher education institutions and faculty within public health programs have a critical role to play when transitioning their students from campus to online teaching formats or vice versa. Careful consideration around curriculum design to ensure it meets the needs of students who are enrolled in a range of learning modalities, such as hybrid, in-person or online, can help maintain a positive learning experience for students (10). Educational institutions may also consider conducting focused studies to better understand how public health curriculum should be designed to best meet these needs, including adopting newer evidence-based pedagogical frameworks to facilitate meaningful online learning (25). Specifically for public health academic programs that offer a majority of courses in an online format, Dabbagh (25) pedagogical framework recommends incorporating a combination of instructional strategies, learning technologies and activities that are tailored for the online environment, to benefit from a triadic interaction between these three concepts to increase student learning experiences. As part of this, it remains important for faculty to continue to play an active and intentional role in the learning environment, be cooperative, constructive and authentic in their course delivery approaches for students to remain engaged in the course (25).

Our study participants indicated a preference for shifting to hybrid academic models as a way to balance work-life commitments which would also reduce mental health concerns. This is consistent with recent studies that aimed to understand whether hybrid learning is effective and in particular, whether in-person presence really matters (26, 27). These authors urge educators in higher education to consider implementing a thoughtful approach to redesigning programs to be hybrid models such as by incorporating training for both faculty and students on the new teaching platforms, such as Canvas or Blackbaud, that will be used to provide instruction. While this may require a cost investment and faculty planning committees from the start, hybrid programs may have a far-reaching impact on students who may otherwise struggle to maintain work-life balance while enrolled in their public health academic program. Students who have other financial obligations may find it easier and more cost-effective to attend classes a few days per week in-person as opposed to daily in-person attendance. It may also simply be much more convenient for the student body overall to enroll in hybrid programs. From a social connectedness and community belonging standpoint, hybrid programs may enable students to develop relationships with their peers and other faculty and participate in campus activities depending on their scheduling preferences rather than around required times when classes are scheduled to be held in-person (28). Therefore, educational practitioners are encouraged to offer hybrid academic programs and establish resource infrastructures that provide students with social and mental wellness support when transitioning to different learning modalities.

### Strengths and limitations

Our study results are not generalizable due to the small sample size of the quantitative component; however, using a phenomenological approach to gather qualitative data provided the research team with deeper insight into student learning experiences while transitioning from campus to online learning. This provides public health academic departments valuable recommendations and insights in the event of future mandated modality shifts in learning environments. Future studies should include a larger sample size that is representative of a wider geographical region and that considers cultural representativeness; future studies should also evaluate hybrid models to determine their feasibility in different higher education contexts and among program faculty.

## Data availability statement

The datasets presented in this article are not readily available because data sharing will affect participant confidentiality. Requests to access the datasets should be directed to haritagroia@gmail.com.

## Ethics statement

The studies involving humans were approved by the San Jose State University Institutional Review Board. The studies were conducted in accordance with the local legislation and institutional requirements. Participants reviewed a consent notice to participate in the study; those opting for an interview provided additional written consent. The participants provided their written informed consent to participate in this study.

## Author contributions

HA: Data curation, Formal analysis, Investigation, Methodology, Software, Visualization, Writing – original draft, Writing – review & editing. RB: Formal analysis, Methodology, Software, Visualization, Writing – review & editing. AC: Conceptualization, Data curation, Investigation, Methodology, Validation, Writing – review & editing. VG: Conceptualization, Project administration, Writing – review & editing. MA: Conceptualization, Funding acquisition, Investigation, Resources, Supervision, Writing – review & editing.

## Appendix 1 Semi-structured interview guide

### Learning experiences

1. Please describe your learning experiences generally [while enrolled in the SJSU MPH program] prior to the pandemic (prior to March 2020)?
a. What were your expectations coming into the program?
b. To what extent did your expectations change during the pandemic?
2. To what extent did going into the pandemic renew or broaden their interest in public health?
3. Did the pandemic bring new insights to how you think about public health issues or how these issues can or should be dealt with?
4. What improvements did you observe, if any, in your capacity to complete the MPH program?
5. Is there anything the department can learn about this experience [the pandemic]?
6. Can you provide more context regarding motivation to do well in school during your time in the pandemic?

### Social connectedness

7. Have you noticed a difference in your levels of social connection pre vs. during the pandemic?
8. How would you describe your current state of maintaining social connection?
a. Have you experienced feelings of isolation and exclusion?
b. What strategies can you use to prevent feelings of isolation and exclusion?

### Mental health

9. What does mental health mean to you?
10. From your perspective, has the COVID-19 pandemic affected your mental health?
a. If Yes—please describe how your mental health has been affected?
b. If No—How have you managed your mental health during the pandemic?
11. How has the transition to online learning affected your mental health? How can SJSU support you during the transition back to in person?
12. What resources have you found to be most beneficial during the pandemic? Why and how did they impact your mental health?

### Barriers

13. Did you experience identity-related barriers to success in the program (e.g., race, ethnicity, gender, sexual orientation) during this time?
a. IF YES: Please provide context to your answer.
b. IF NO: Did you notice identity-related facilitators?
14. Did you experience any other barriers that prevented you from having success in the program?
a. IF YES: Please provide context to your answer.
b. IF NO: Did you notice other facilitators?
